# *In ovo* galactooligosaccharides and sodium butyrate induce segment-specific changes in gut microbiota of broiler chickens

**DOI:** 10.1016/j.psj.2026.107276

**Published:** 2026-06-10

**Authors:** S. Knaga, M.Z. Akram, A. Bełdowska-Krężel, E. Pietrzak, A. Dunisławska

**Affiliations:** a Department of Animal Biotechnology and Genetics, Bydgoszcz University of Science and Technology, 28 Mazowiecka St., Bydgoszcz, 85-084, Poland; b Nutrition and Animal-Microbiota Ecosystems Laboratory, Department of Biosystems, KU Leuven, Heverlee, 3000, Belgium

**Keywords:** Broiler, Intestinal microbiota, Early programming, Prebiotic, Short-chain fatty acid

## Abstract

Early-life in ovo interventions are increasingly explored as tools to modulate poultry gut ecosystems and intestinal function. However, high-throughput evidence describing ileal and cecal microbiota responses following embryonic exposure remains limited. This study evaluated whether a single in ovo administration of a prebiotic (galactooligosaccharides) or a short-chain fatty acid donor (sodium butyrate) resulted in differences in gut microbial communities of broiler chickens at day 42 of age. On embryonic day 12, 1,068 fertile Ross 308 eggs were assigned to four treatments: non-injected control, saline-injected control, galactooligosaccharides (3.5 mg/0.2 mL), or sodium butyrate (0.3%/0.2 mL). Birds were reared to day 42, and ileal and cecal digesta were collected from 10 birds per group. Full-length 16S ribosomal RNA gene sequencing was used to characterize microbial composition and diversity. Galactooligosaccharides increased richness and diversity while maintaining dominance of *Lactobacillus* and promoting *Romboutsia* and unclassified Peptostreptococcaceae. Sodium butyrate induced a stronger restructuring of the microbiota, accompanied by reduced *Lactobacillus* abundance and enrichment of multiple Clostridia-associated genera together with representatives of Bacteroidota; community structure differed among treatments. In the cecum, galactooligosaccharides increased diversity and promoted expansion of taxa affiliated with Lachnospiraceae and Ruminococcaceae, including *Faecalibacterium, Blautia, Barnesiella*, and *Sporobacter*. In contrast, sodium butyrate reduced microbial diversity while enriching several Clostridia-associated taxa. Discriminant analysis identified distinct marker taxa in the ileum and cecum. In the ileum, sodium butyrate showed a broader range of discriminant taxa, whereas in the cecum most discriminant markers belonged to the class Clostridia. These findings demonstrate that galactooligosaccharides and sodium butyrate differentially modulate microbial communities in the ileum and cecum, highlighting the distinct effects of prebiotic and postbiotic in ovo interventions on gut microbiota composition.

## Introduction

Early nutritional interventions during the embryonic period represent a promising strategy for programming poultry health and productivity throughout the entire production cycle. Among the substances employed in *in ovo* strategies, prebiotics and postbiotics are of particular interest, as they influence the developing gastrointestinal tract prior to hatching.

Galactooligosaccharides (GOS) are among the most extensively studied prebiotics in poultry nutrition. They selectively stimulate the proliferation of beneficial intestinal bacteria, particularly members of the genera *Lactobacillus* and *Bifidobacterium*, while limiting colonization by potentially pathogenic microorganisms (Patterson and Burkholder, 2003; Hajati and Rezaei, 2010; Slawinska et al., 2019). The chicken gastrointestinal microbiota, which is predominantly composed of bacteria belonging to the phyla Firmicutes and Bacteroidetes, plays a pivotal role in immune regulation, metabolic processes, and maintenance of intestinal barrier integrity (Wei et al., 2013; Oakley et al., 2014; Clavijo and Flórez, 2018). Therefore, modulation of the microbiota during early development may have long-term physiological consequences.

Administration of GOS on day 12 of incubation enables stimulation of the developing gastrointestinal tract and promotes early colonization by commensal microbiota (Pedroso et al., 2016; Roto et al., 2016). This procedure has been demonstrated to be safe for the incubation process, without adversely affecting hatchability, while improving chick quality and increasing the relative abundance of *Lactobacillus* and *Bifidobacterium* species (Mangan et al., 2025b). Moreover, GOS delivered in ovo has been associated with improved small intestinal histomorphology, including increased villus height and absorptive surface area (Madej et al., 2015; Bogucka et al., 2016). Studies have also demonstrated its influence on the development of gut-associated lymphoid tissue (GALT) (Sławińska et al., 2014; Madej and Bednarczyk, 2016). Furthermore, GOS administration has been linked to long-term alterations in the expression of genes involved in immune responses and adaptation to environmental stress (Slawinska et al., 2016; Pietrzak et al., 2020). Recent findings indicate that in ovo GOS administration may enhance tissue antioxidant capacity. In addition, GOS may regulate the expression of barrier-related genes and receptors involved in short-chain fatty acid metabolism. These effects were observed without negative changes in plasma metabolic parameters (Mangan et al., 2025a).

Sodium butyrate (**SB**), the sodium salt of butyric acid classified as a postbiotic, is one of the principal short-chain fatty acids involved in maintaining intestinal homeostasis. Supplementation with SB, either via feed or in ovo administration, has been shown to modulate the expression of genes associated with intestinal barrier integrity, including *TJP1* (ZO-1), *CLDN1* (claudin-1), *OCLN* (occludin), and mucin-encoding genes such as *MUC2* (Bortoluzzi et al., 2017; Melaku et al., 2024). In addition, SB has been reported to regulate immune responses and enhance antioxidant capacity (Bełdowska et al., 2024; Akram et al., 2024). Additionally, SB and its protected forms have demonstrated the ability to improve intestinal villus morphology, enhance nutrient digestibility, and reduce the abundance of potentially pathogenic bacteria (Makowski et al., 2022; Deng et al., 2023; Melaku et al., 2024). The productive response to SB appears to depend on its form and dosage; in *in ovo* models, a modulatory effect has generally been observed rather than a consistently growth-promoting response (Dunisławska et al., 2024).

Interventions involving both GOS and SB are consistent with the concept of early microbiome modulation. According to this concept, changes in the intestinal environment during embryonic development may influence intestinal barrier function, immune responsiveness, and host metabolic regulation later in life.

However, current evidence remains fragmented, as most studies have focused on early posthatch stages, selected bacterial taxa, or a single intestinal segment, limiting the assessment of microbiota responses across different intestinal compartments and developmental stages following *in ovo* interventions.

Importantly, inconsistent findings across studies may also arise from differences in sampling location along the gastrointestinal tract, which is rarely considered as a primary factor shaping microbial responses.

Microbial responses may differ between intestinal compartments. The ileum primarily fulfills digestive and absorptive functions, whereas the ceca constitute the principal site of bacterial fermentation and short-chain fatty acid (**SCFA**) production. Physiological and environmental differences between these intestinal segments may therefore result in distinct colonization patterns and differential responsiveness to early nutritional interventions. Consequently, simultaneous evaluation of the ileal and cecal microbiota is essential for understanding how early-life dietary interventions are associated with microbial community composition across intestinal compartments.

The aim of the present study was to test the hypothesis that *in ovo* administration of a prebiotic (GOS) or a postbiotic (SB) was associated with segment-specific differences in the microbiota of the ileum and ceca at day 42 of age.

## Materials and methods

### Ethical statement

The study was conducted in accordance with applicable animal welfare regulations, including Directive 2010/63/EU of 22 September 2010 and the Polish Act of 15 January 2015 on the protection of animals used for scientific or educational purposes. In ovo administration was performed on embryonic day 12 by injection into the air cell of fertile eggs. Biological material was collected post mortem. According to the applicable regulations, the study did not involve procedures requiring authorization by a local ethics committee for animal experimentation.

### Experimental design and animal management

Hatching eggs were obtained from a Ross 308 broiler parental flock (Drobex, Makowiska, Poland). A total of 1,200 eggs (mean weight 70.80 ± 1.32 g; 10 trays) were placed in a commercial incubator (Jarson, Gostyń, Poland). Incubation was carried out at 37.7°C with relative humidity maintained between 55 and 60% and ventilation set at 50%. During incubation, eggs were positioned on automatically turning trays and rotated by 45° from the horizontal plane at 1-hour intervals. Candling was performed on day 7 to assess fertility and embryonic viability. Unfertilized eggs and those containing nonviable embryos were removed (early embryonic mortality up to day 7: 2.58%). A second candling was conducted on day 11, resulting in the removal of additional nonviable embryos (cumulative mortality up to day 11: 1.33%). Fertility rate at day 7 reached 89% of the initially set eggs.

On day 12 of incubation, 1,068 confirmed fertile eggs were randomly assigned to four experimental groups and subjected to *in ovo* procedures. Following treatment allocation and injection, eggs from all experimental groups were distributed across incubator trays and maintained in the same incubator under identical incubation conditions to minimize potential positional effects within the incubation system. Injection sites were sealed immediately after treatment to prevent contamination and moisture loss. The first group served as a non-injected control (**C**). The second group received an injection of sterile physiological saline and constituted the injected control (**CI**). In the third group, galactooligosaccharides (**GOS**; Clasado Ltd., Reading, UK) were administered. The injection solution was prepared under aseptic conditions by dissolving 3.5 mg of GOS in 0.2 mL of sterile saline. The fourth group received sodium butyrate (**SB**; sodium salt of butyric acid; molecular weight 110.09; Merck Life Science, Warsaw, Poland). A 0.3% SB solution was prepared in physiological saline, and 0.2 mL was injected per egg. All injections were delivered into the air cell following the *in ovo* protocol previously described by Dunisławska et al. (2023).

Following injection, incubation continued under unchanged environmental conditions. On day 18, eggs were candled again and transferred to a hatcher (Jarson, Gostyń, Poland). During transfer, eggs from all treatment groups were again randomly distributed within the hatcher. All eggs were hatched in the same hatching unit under identical environmental conditions. Hatching conditions were maintained at 37.5°C, 70% relative humidity, and 80% ventilation. The chicks hatched on d 21.

After hatching, one-day-old male chicks with an average body weight of 49.66 ± 8.19 g were selected for further rearing. A total of 84 birds per treatment group (C, CI, GOS, SB) were transferred to the experimental broiler facility. Chickens were housed in stainless-steel mesh floor pens at a stocking density of 33 kg/m². Each treatment consisted of 7 replicates, with 12 birds per pen. Pens were bedded with chopped wheat straw. Continuous access to fresh water was provided via two nipple drinkers per pen, and feed was supplied in wall-mounted feeders. Ambient temperature in the broiler house was gradually reduced from 30°C on day 1 to 21°C by week 4, while relative humidity was maintained at approximately 60%. Environmental parameters were monitored and adjusted based on both instrumental measurements and behavioral observations of the birds, in accordance with previously described management procedures (Biesek et al., 2023). The rearing period lasted 42 days. Feeding was divided into three phases: starter diet (day 1–14), grower diet (day 15–35), and finisher diet (day 36–42). All diets were commercially formulated (Golpasz, De Heus, Golub-Dobrzyń, Poland) and provided ad libitum. Diets were isocaloric and isonitrogenous, formulated in accordance with poultry nutrition recommendations (Smulikowska and Rutkowski, 2019).

Chemical composition of the feeds was determined using near-infrared spectroscopy with a FoodScan device (FOSS, Hillerød, Denmark), according to ISO 12099:2017.

The chemical composition of the experimental diets is presented in Table 1. Values are expressed as g/kg of feed (dry matter) or g/kg of dry matter and represent the mean of six analytical replicates per dietary phase; manufacturer data were verified by laboratory analysis (Dunisławska et al., 2024).

**Table 1:** Chemical composition of the commercial diet for broiler chickens.

Ingredient	Commercial diet		
	Starter	Grower	Finisher
Dry matter (g/kg of feed)	887.23	883.60	880.73
Crude ash (g/kg of DM)	61.71	64.89	52.55
Total protein (g/kg of DM)	219.16	205.37	183.05
Crude fat (g/kg of DM)	46.36	42.91	34.23
Crude fiber (g/kg of DM)	48.15	42.87	44.47

### Sample collection

On day 42 of rearing, broilers were individually weighed and 10 birds per treatment group (n = 40 in total) were selected for further analyses. Individuals with body weights close to the group mean were chosen to ensure representative sampling. Prior to slaughter, feed was withdrawn for 8 hours while water remained available. All birds were subjected to the same 8-hour feed withdrawal period before sample collection, irrespective of treatment group. All procedures complied with European Union legislation on animal welfare, including Council Regulation (EC) No 1099/2009 and Directive 2010/63/EU. Birds were humanely stunned by a controlled percussive blow to the head, inducing immediate unconsciousness. Subsequently, decapitation was performed by severing the head at the atlanto-occipital joint, allowing rapid exsanguination of the carcass.

During slaughter, digesta samples from the ileum and ceca were aseptically collected into sterile 5-mL tubes. Samples were immediately snap-frozen on dry ice and transported to the laboratory, where they were stored at −80°C until bacterial DNA extraction. Total bacterial genomic DNA was extracted from approximately 25 mg of ileal and cecal digesta using a Stool DNA Purification Kit (EURx, Poland), according to the manufacturer’s instructions. DNA extraction included a mechanical bead-beating step using glass beads and vortexing at 2500 rpm for 10 min following thermal and chemical lysis. DNA concentration and purity were determined with a NanoDrop 2000 spectrophotometer (Thermo Scientific, USA). Nucleic acid integrity was evaluated by electrophoresis on a 2% agarose gel. Purified DNA samples were subsequently stored at −80°C until further downstream analyses.

### Libraries preparation and sequencing

Extracted bacterial DNA was subjected to full-length 16S rRNA gene sequencing using the 16S Barcoding Kit (SQK-16S114.24; Oxford Nanopore Technologies, Oxford, UK). PCR amplification targeted the complete V1–V9 hypervariable regions with universal primers 27F (5′-AGAGTTTGATCMTGGCTCAG-3′) and 1492R (5′-CGGTTACCTTGTTACGACTT-3′).

Each PCR reaction consisted of 25 µL LongAmp Hot Start Taq 2 × Master Mix (New England Biolabs, Ipswich, MA, USA), 10 µL of barcode solution, and 15 µL of DNA template (10 ng). Thermal cycling conditions included an initial denaturation at 95°C for 1 min, followed by 25 cycles of denaturation at 95°C for 20 s, annealing at 55°C for 30 s, and extension at 65°C for 120 s, with a final extension step at 65°C for 5 min. The concentration of barcoded PCR products was initially assessed using the Qubit dsDNA HS Assay Kit (Thermo Fisher Scientific, Waltham, MA, USA) without prior purification. Amplicons were subsequently purified twice using AMPure XP beads (Beckman Coulter, Brea, CA, USA) with 80% ethanol washes. Cleaned libraries were quantified by fluorometry (Qubit 4.0 fluorometer, dsDNA HS Assay Kit) and pooled in equimolar ratios. A total of 100 ng of pooled libraries in 11 µL was loaded onto an R10.4.1 flow cell (Oxford Nanopore Technologies, Oxford, UK) installed in a MinION Mk1B device. The raw metagenomic sequencing data generated in this study have been deposited in the NCBI under BioProject accession number PRJNA1436604 and are publicly accessible.

### Bioinformatic analysis

Raw sequencing reads were first subjected to demultiplexing and basecalling using Dorado (v. 0.9.1; Oxford Nanopore Technologies). Adapter trimming and quality filtering were performed with NanoFilt (v. 2.8.0). Reads shorter than 200 bp and those with a mean quality score below Q10 were discarded. High-quality reads were retained in FASTQ format for downstream analysis.

Quality control included filtering based on read length and quality thresholds and removal of low-quality reads to ensure reliability of downstream analyses.

Microbiota analysis was conducted in QIIME 2 (v. 2024.10). Sequences were dereplicated using the vsearch plugin, followed by de novo operational taxonomic unit (OTU) clustering at 97% sequence identity. Representative sequences from each OTU were assigned taxonomy against the SILVA reference database (release 138) using the QIIME2 (Bolyen et al., 2019) vsearch classifier.

Alpha diversity indices, including Observed richness, Shannon diversity, and Simpson diversity, were calculated after rarefaction of the OTU table to the minimum sequencing depth across samples using R (v. 4.2.3). Differences in alpha diversity between experimental groups were evaluated using the Kruskal–Wallis test, followed by pairwise Wilcoxon rank-sum tests for post hoc comparisons. P values from all pairwise comparisons were adjusted for multiple testing using the Benjamini–Hochberg false discovery rate (FDR) procedure.

Beta diversity was assessed based on Bray–Curtis dissimilarity matrices and visualized using principal coordinate analysis (**PCoA**). The impact of treatments on β-diversity was assessed using a non-parametric permutational multivariate analysis of variance (**PERMANOVA**). This analysis was performed with the adonis2 function from the vegan package (v. 2.6.4), applying 999 permutations to test for multivariate differences among groups. Beta dispersion was assessed using PERMDISP in R (v. 4.2.3) to test whether within-group variability differed between samples. A Bray-Curtis distance matrix was calculated from the phyloseq object, matching the distance metric used for the PCoA and PERMANOVA analyses. Group dispersions were estimated as the distances of samples to their respective group centroids using betadisper, and significance was tested by permutation using permutest with 9,999 permutations.

Differentially abundant taxa were identified using linear discriminant analysis effect size (**LEfSe** - Linear Discriminant Analysis Effect Size) implemented in R. LEfSe was performed as a global multi-group comparison using the run_lefse function on genus-level taxa from the phyloseq object. Counts were normalized using counts per million (norm = "CPM"), and group differences were tested using the Kruskal-Wallis test with a significance threshold of p < 0.05. Taxa passing the Kruskal-Wallis test were further evaluated by linear discriminant analysis, and features with an LDA score ≥ 3 were retained as discriminant taxa. FDR correction was applied after extracting the LEfSe marker table by adjusting the LEfSe pvalue column across all tested genus-level taxa using the Benjamini-Hochberg method. To make our differential abundance analysis more robust, we also performed ANCOM-BC2 using the ANCOMBC R package (v. 2.0.3). The analysis was conducted on raw count data from the phyloseq object at the genus level, with group included as the fixed-effect variable. P-values were adjusted using the Benjamini-Hochberg FDR method, and structural zero detection and negative lower-bound correction were enabled. Differentially abundant genera identified by ANCOM-BC2 were visualized based on ANCOM-BC2 log-fold-change estimates.

## Results

A total of 5,296,922 reads obtained from 80 samples were included in the microbiota analysis, resulting in an average of 66,212 reads per sample with a standard deviation of 85,356 reads (range: minimum = 8,609 and maximum = 347,599). To standardize the α-diversity analysis, the sample with the lowest sequencing depth was used as the cut-off threshold for rarefying all samples.

### Intestinal microbial diversity structure

**Fig. 1:**
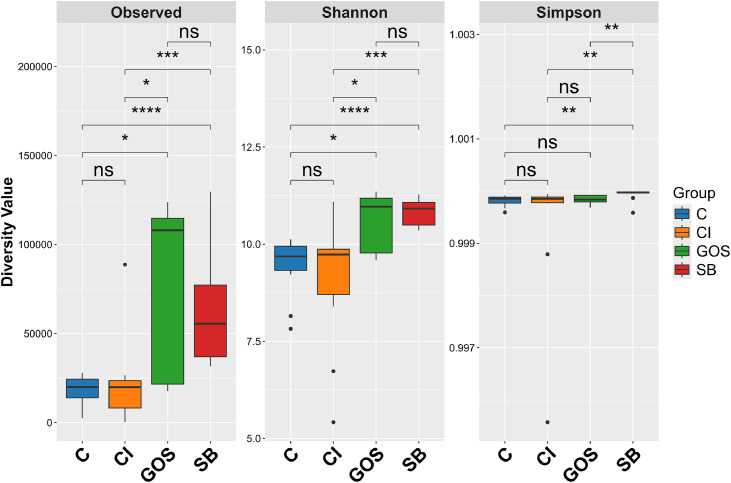
Alpha diversity of the ileal microbiota following *in ovo* administration of bioactive compounds.

**Fig. 2:**
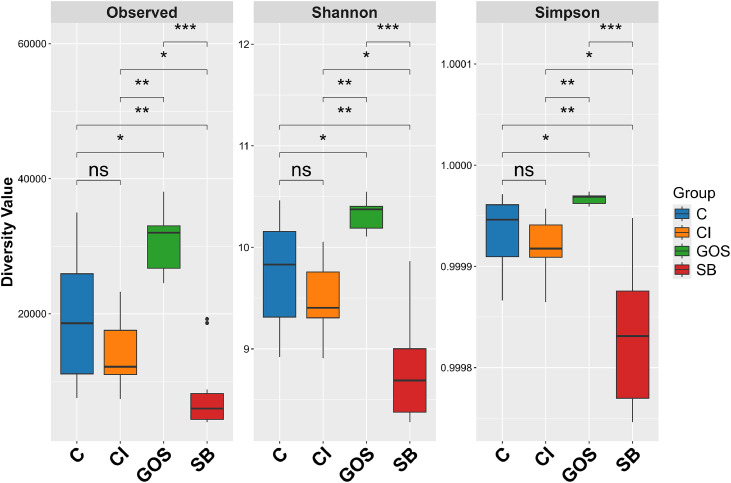
Alpha diversity of the cecal microbiota following *in ovo* administration of bioactive compounds.

Alpha diversity analysis revealed significant effects of GOS and SB on microbial diversity in both intestinal segments (Fig. 1, Fig. 2). In the ileum and cecum, the highest observed richness and Shannon and Simpson indices were recorded in GOS, which differed significantly from C and CI for observed richness and Shannon index in both segments. No differences in the Simpson index were detected between GOS and the controls in the ileum, whereas in the cecum Simpson values were significantly higher in GOS than in C and CI. Sodium butyrate differed significantly from C and CI for all alpha diversity indices in both segments. However, the response differed between intestinal segments. In the ileum, SB increased observed richness as well as Shannon and Simpson diversity indices compared with the control groups. In contrast, all three indices were significantly lower in the cecum. Differences between GOS and SB were limited to the Simpson index in the ileum, while in the cecum SB showed lower values of all alpha diversity indices compared with GOS. No effect of saline injection alone (C vs CI) was observed in either intestinal segment.

C - control group; CI - saline-injected control group; GOS - galactooligosaccharide group; SB - sodium butyrate group. Differences between groups are indicated as follows: * P < 0.05; ** P < 0.01; *** P < 0.001; **** P < 0.0001; ns - not significant.

C - control group; CI - saline-injected control group; GOS - galactooligosaccharide group; SB - sodium butyrate group. Differences between groups are indicated as follows: * P < 0.05; ** P < 0.01; *** P < 0.001; **** P < 0.0001; ns - not significant.

**Fig. 3:**
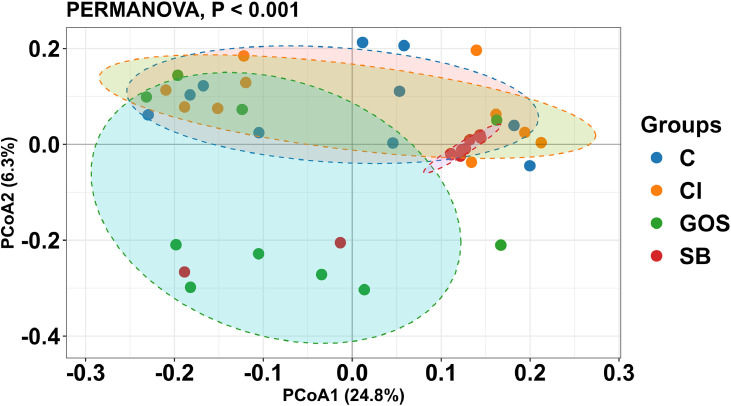
Principal coordinates analysis (PCoA) of ileal microbial communities based on Bray–Curtis dissimilarity.

**Fig. 4:**
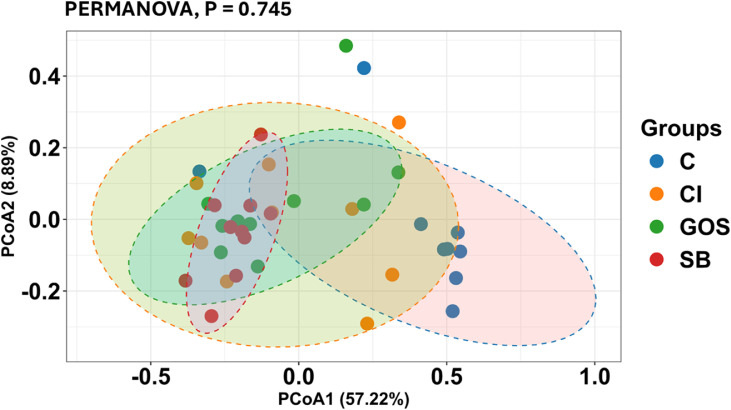
Principal coordinates analysis (PCoA) of cecal microbial communities based on Bray–Curtis dissimilarity.

**Fig. 5:**
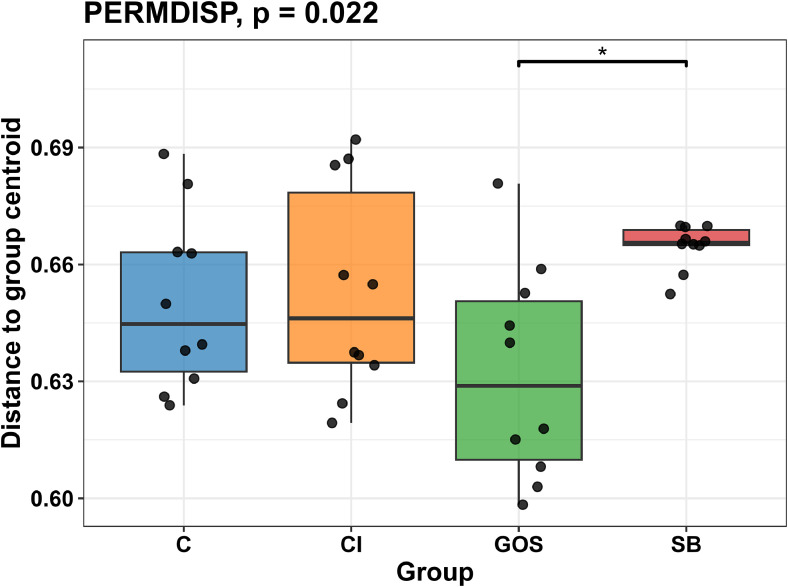
Homogeneity of multivariate dispersion (PERMDISP) of ileal microbial communities based on Bray–Curtis dissimilarity.

**Fig. 6:**
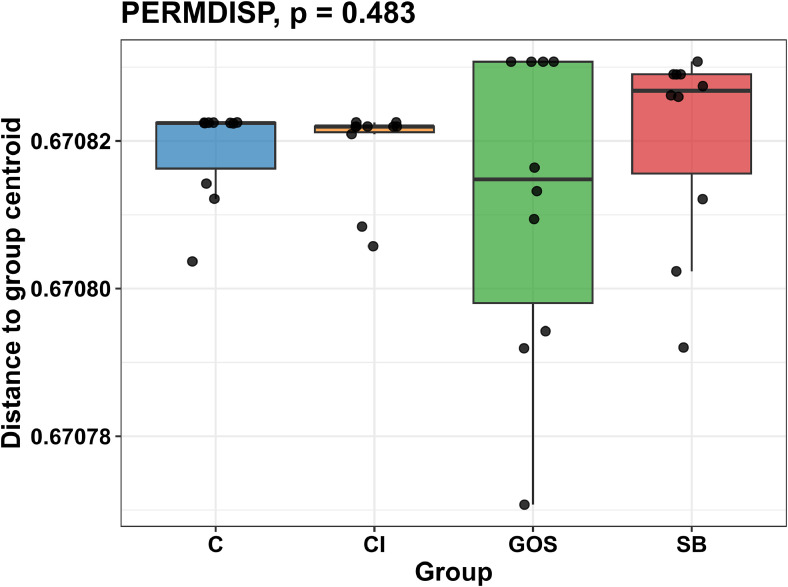
Homogeneity of multivariate dispersion (PERMDISP) of cecal microbial communities based on Bray–Curtis dissimilarity.

Bray-Curtis-based beta diversity analysis demonstrated significant differences in microbial community structure among groups in the ileum (Fig. 3; PERMANOVA, p < 0.001, R^2^ = 0.086), whereas no significant group effect was detected in the caecum (Fig. 4; PERMANOVA, p = 0.745, R^2^ = 0.077). In the ileum, C and CI largely overlapped, GOS occupied an intermediate position, and SB showed partial separation from the other groups. PERMDISP analysis indicated significant differences in within-group dispersion among ileal groups (F = 3.63, p = 0.0218), with post hoc testing showing a significant difference between SB and GOS (adjusted p = 0.0126), but not between SB and C. Therefore, ileal PERMANOVA results should be interpreted with consideration of unequal group dispersion. In the caecum, PERMDISP analysis was not significant (F = 0.84, p = 0.4834), indicating no detectable differences in within-group dispersion among groups (Figs. 5 and 6).

C - control group; CI - saline-injected control group; GOS - galactooligosaccharide group; SB - sodium butyrate group. The percentage of variation explained by each axis is indicated in parentheses. Group differences were tested using PERMANOVA (P < 0.001).

C - control group; CI - saline-injected control group; GOS - galactooligosaccharide group; SB - sodium butyrate group. The percentage of variation explained by each axis is indicated in parentheses. Group differences were tested using PERMANOVA (P = 0.745).

### Taxonomic structure of dominant gut genera

**Fig. 7:**
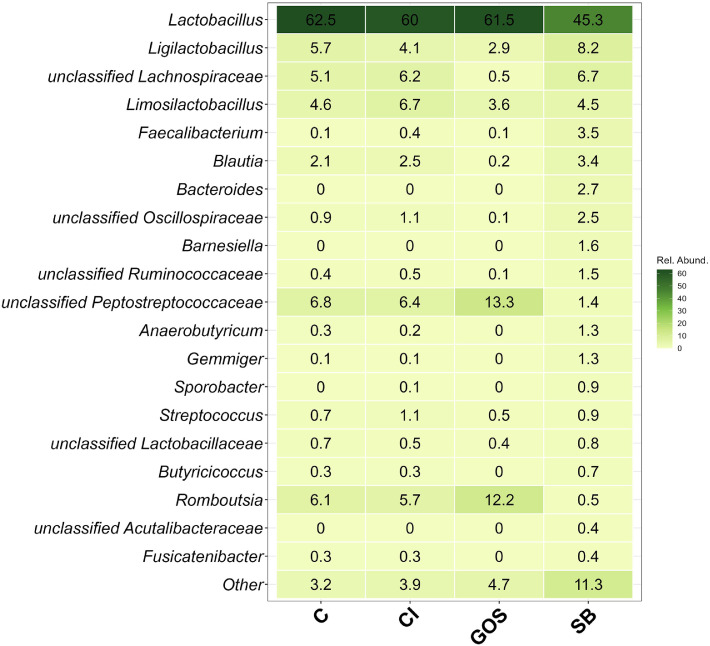
Relative abundance of dominant ileal bacterial genera after *in ovo* administration of bioactive compounds.

The ileal microbiota was dominated by Lactobacillaceae (phylum Firmicutes), including *Lactobacillus, Ligilactobacillus*, and *Limosilactobacillus* (Fig. 7). *Lactobacillus* was most abundant in C, CI, and GOS (60.0–62.5%) and lower in SB (45.3%), whereas *Ligilactobacillus* was enriched in SB (8.2%) relative to the remaining groups. Peptostreptococcaceae (class Clostridia), represented by unclassified Peptostreptococcaceae and *Romboutsia*, reached their highest abundance in GOS (13.4% and 12.2%), moderate levels in C and CI (∼6%), and lower levels in SB (1.4% and 0.5%). In contrast, SB showed increased abundance of several Clostridia-affiliated genera within Lachnospiraceae, Ruminococcaceae, and Oscillospiraceae, including *Faecalibacterium, Blautia, Anaerobutyricum, Butyricicoccus*, and *Gemmiger*, which remained ≤1% in C, CI, and GOS. Members of Bacteroidota, namely *Bacteroides* (2.7%) and *Barnesiella* (1.6%), were detected exclusively in SB. C, CI, and GOS retained a Lactobacillaceae-dominated profile, whereas GOS was characterized by higher relative abundance of Peptostreptococcaceae-related taxa and SB by increased abundance of Clostridia-affiliated genera together with the presence of Bacteroidota.

The heatmap shows the relative abundance (%) of major bacterial genera in control (C), saline-injected control (CI), galactooligosaccharide (GOS), and sodium butyrate (SB) groups.

**Fig. 8:**
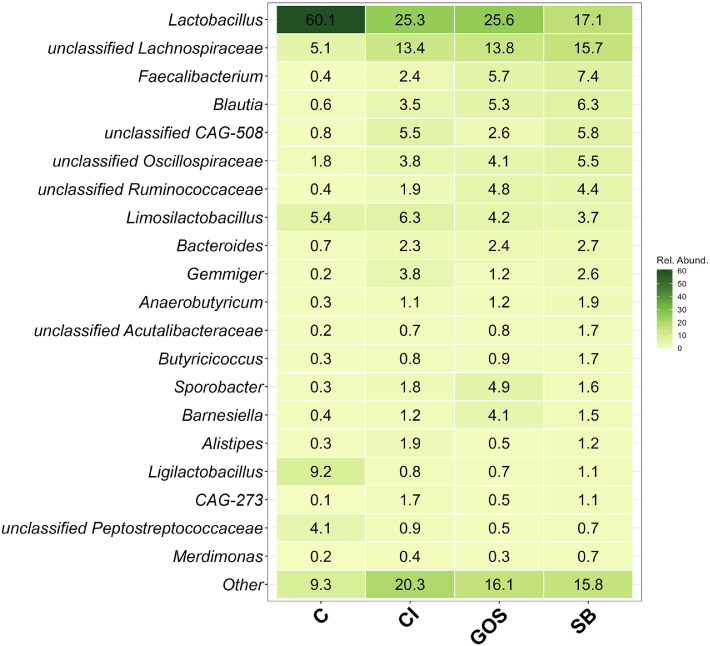
Relative abundance of dominant cecal bacterial genera after *in ovo* administration of bioactive compounds.

In the cecum, C was dominated by Lactobacillaceae, including *Lactobacillus, Ligilactobacillus*, and *Limosilactobacillus* (Fig. 8). *Lactobacillus* reached its highest abundance in C (60.1%) and was lower in CI and GOS (∼25%) and SB (17.1%), while *Ligilactobacillus* was also most abundant in C (9.2%) and remained low (0.7–1.1%) in the other groups. Compared with C, the remaining groups exhibited increased abundance of taxa affiliated with Lachnospiraceae, Ruminococcaceae, and Oscillospiraceae (class Clostridia). Unclassified Lachnospiraceae increased from 5.1% in C to 13.4–15.7% in CI, GOS, and SB. Similar increases were observed for *Faecalibacterium* (0.4% to 7.4%) and *Blautia* (0.6% to 6.3%), as well as for *Anaerobutyricum, Butyricicoccus*, and *Gemmiger*. Members of Bacteroidota, including *Bacteroides, Barnesiella*, and *Alistipes*, were present in all groups but were more abundant in CI, GOS, and SB than in C. In contrast, unclassified Peptostreptococcaceae were highest in C (4.1%) and lower in the remaining groups (0.5–0.9%). C exhibited a Lactobacillaceae-dominated structure, whereas CI, GOS, and SB showed higher relative abundance of Clostridia-affiliated taxa and Bacteroidota.

The heatmap shows the relative abundance (%) of major bacterial genera in control (C), saline-injected control (CI), galactooligosaccharide (GOS), and sodium butyrate (SB) groups.

### Group-associated microbial signatures

**Fig. 9:**
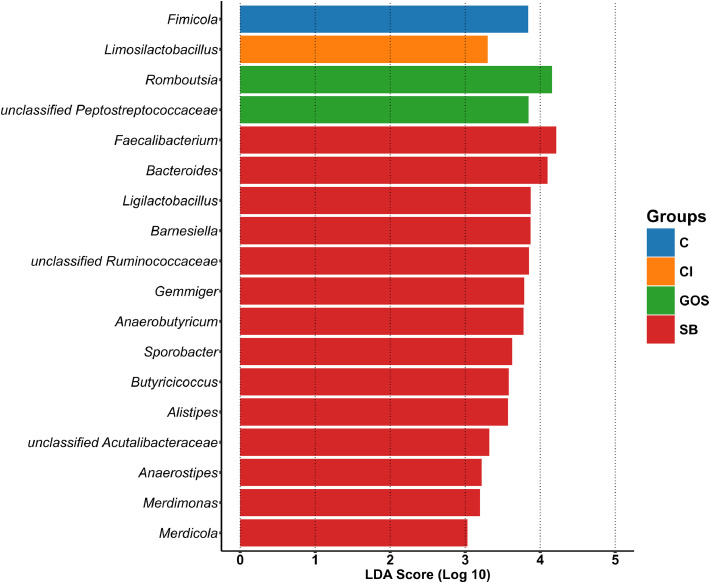
Differentially enriched ileal bacterial genera identified by LEfSe after *in ovo* administration of bioactive compounds. Linear discriminant analysis (LDA) scores indicate taxa with significant differences among control (C), saline-injected control (CI), galactooligosaccharide (GOS), and sodium butyrate (SB) groups. LEfSe analysis was performed using a linear discriminate analysis (LDA) score threshold of ≥ 3.0. P-values were adjusted for multiple testing using the Benjamini-Hochberg false discovery rate (FDR) method. Bar colors indicate the group in which each discriminant genus was enriched.

**Fig. 10:**
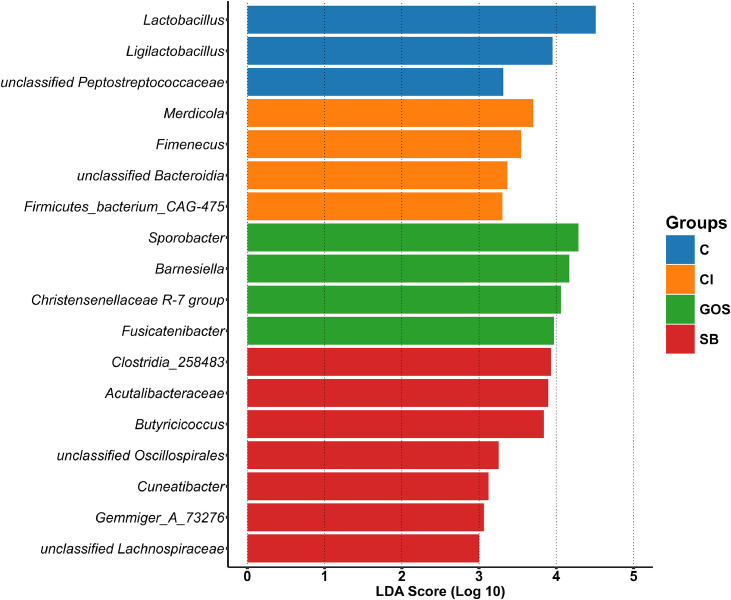
Differentially enriched cecal bacterial genera identified by LEfSe after *in ovo* administration of bioactive compounds. Samples were obtained from chickens in control (C), saline-injected control (CI), galactooligosaccharide (GOS), and sodium butyrate (SB) groups. LEfSe analysis was performed using a linear discriminate analysis (LDA) score threshold of ≥ 3.0. P-values were adjusted for multiple testing using the Benjamini-Hochberg false discovery rate (FDR) method. Bar colors indicate the group in which each discriminant genus was enriched.

LEfSe analysis was performed to identify bacterial taxa that significantly differentiated the microbial communities among the analyzed groups in the ileum (Fig. 9) and cecum (Fig. 10). In both intestinal segments, 18 taxa met the predefined significance thresholds (LDA ≥ 3.0; FDR < 0.05).

In the ileum, discriminant taxa were unevenly distributed across groups. In C and CI, a single discriminant taxon was identified in each group, namely *Fimicola* and *Limosilactobacillus*, respectively. In GOS, the discriminant taxa included *Romboutsia* and unclassified Peptostreptococcaceae.

The highest number of discriminant taxa was observed in SB. These taxa belonged to two major phylogenetic lineages. The first comprised members of the class Clostridia (phylum Bacillota/Firmicutes), including the genera *Faecalibacterium, Gemmiger, Anaerobutyricum, Butyricicoccus*, and *Anaerostipes*, as well as unclassified taxa assigned to the families Ruminococcaceae and Acutalibacteraceae and to the order Oscillospirales. The second lineage consisted of taxa belonging to the phylum Bacteroidota, represented by *Bacteroides, Barnesiella*, and *Alistipes*. In addition, SB was characterized by several individual discriminant taxa within Bacillota, including *Ligilactobacillus, Sporobacter, Merdimonas*, and *Merdicola*. Overall, SB exhibited the most extensive discriminant taxonomic profile, whereas C and CI were characterized by a minimal number of differentiating taxa and GOS by a limited set of markers.

In the cecum, discriminant taxa were more evenly distributed among groups. In C, the markers included *Lactobacillus, Ligilactobacillus*, and unclassified Peptostreptococcaceae. In CI, the identified discriminant taxa were *Merdicola, Fimenecus*, unclassified Bacteroidia, and *Firmicutes bacterium CAG-475*. In GOS, the discriminant taxa comprised *Sporobacter, Barnesiella*, Christensenellaceae R-7 group, and *Fusicatenibacter*.

In SB, all identified discriminant taxa belonged to the class Clostridia and represented multiple taxonomic levels: class (*Clostridia_258483*), order (unclassified Oscillospirales), families (Acutalibacteraceae and unclassified Lachnospiraceae), and genera (*Butyricicoccus, Cuneatibacter*, and *Gemmiger_A_73276*). In contrast to the ileum, each group in the cecum was characterized by a distinct set of discriminant taxa, without a clear predominance of any single group in terms of marker abundance.

To make the differential abundance analysis more robust, we additionally performed ANCOM-BC2. Overall, ANCOM-BC2 identified a largely similar set of discriminative genera and higher-level taxa associated with the experimental groups as observed with LEfSe, supporting the consistency of the group-associated microbial signatures.

**Fig. 11:**
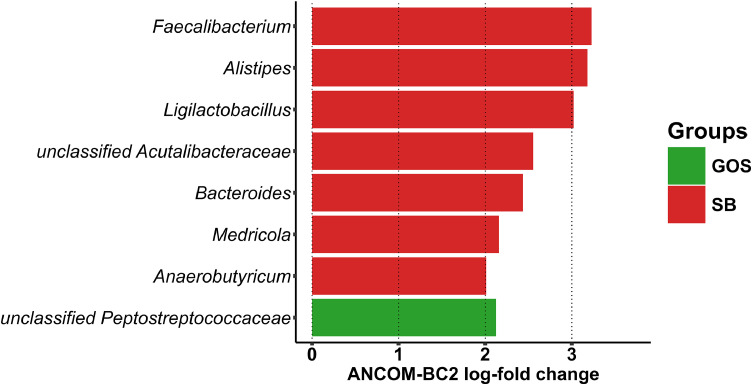
Differentially abundant taxa identified by ANCOM-BC2 in the ileum. Bars represent the absolute ANCOM-BC2 log-fold-change effect size for each taxon, and colors indicate the group with which each taxon was associated. P-values were adjusted for multiple testing using the Benjamini-Hochberg false discovery rate (FDR) method.

In the ileum, ANCOM-BC2 identified eight differentially abundant taxa (Fig. 11). The GOS group was associated with increased abundance of unclassified Peptostreptococcaceae. The remaining differentially abundant taxa were associated with SB. These included *Anaerobutyricum, Merdicola, Bacteroides*, unclassified *Acutalibacteraceae, Ligilactobacillus, Alistipes*, and *Faecalibacterium*.

**Fig. 12:**
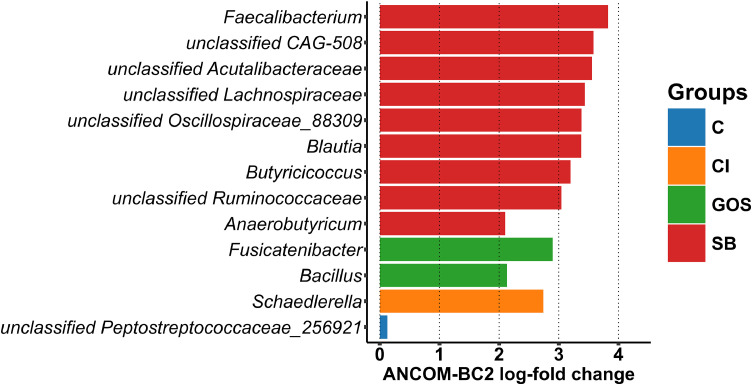
Differentially abundant taxa identified by ANCOM-BC2 in the cecum. Bars represent the absolute ANCOM-BC2 log-fold-change effect size for each taxon, and colors indicate the group with which each taxon was associated. P-values were adjusted for multiple testing using the Benjamini-Hochberg false discovery rate (FDR) method.

In the cecum, ANCOM-BC2 identified thirteen differentially abundant taxa (Fig. 12). CI was associated with *Schaedlerella*, while GOS was associated with *Bacillus* and *Fusicatenibacter*. The majority of differentially abundant taxa were associated with SB, including *Anaerobutyricum*, unclassified Ruminococcaceae, *Butyricicoccus, Blautia*, unclassified Oscillospiraceae_88309, unclassified Lachnospiraceae, unclassified Acutalibacteraceae, unclassified CAG-508, and *Faecalibacterium*. Unclassified Peptostreptococcaceae showed a weak association with C group.

## Discussion

### Impact of the In ovo injection procedure on gut microbial composition

To assess the impact of the *in ovo* injection procedure per se, non-injected controls (C) were compared with saline-injected controls (CI). In the ileum, no significant differences were observed between C and CI in alpha diversity (Observed, Shannon, Simpson) or beta diversity. The dominant taxa remained comparable in both groups, indicating that the injection procedure did not measurably affect ileal microbiota composition.

In contrast, in the cecum, modest taxonomic differences were observed between C and CI despite the absence of significant differences in alpha diversity indices. The CI group showed reduced relative dominance of *Lactobacillus* and increased representation of fermentative taxa affiliated with Lachnospiraceae and Ruminococcaceae, as well as *Faecalibacterium, Blautia*, and selected Bacteroidota. These findings suggest that the injection procedure may influence taxonomic proportions in the cecum without altering overall richness or evenness. This differential response between intestinal compartments should be considered when interpreting the effects of bioactive compounds administered *in ovo*.

### Effect of in ovo GOS administration on the structure and functional potential of the gut microbiota

*In ovo* administration of GOS on day 12 of incubation resulted in differences in gut microbiota composition observed at 42 days of age in broiler chickens. The magnitude and nature of these changes differed between the ileum and cecum. The GOS-associated microbiota profile in the ileum was characterized by increased richness while maintaining dominance of *Lactobacillus*, suggesting selective stimulation of additional bacterial taxa without major restructuring of the core community. The increased abundance of *Romboutsia* and unclassified members of the family Peptostreptococcaceae further supports this interpretation. In particular, the consistent identification of Peptostreptococcaceae in differential abundance analyses suggests that this family may represent one of the bacterial groups most responsive to embryonic GOS administration. A similar pattern of moderate microbiota modulation following dietary GOS supplementation was reported by Yang et al. (2022) and Teague et al. (2023); however, their studies primarily focused on the early posthatch period rather than microbiota differences detected at market age following embryonic exposure. The persistence of *Lactobacillus* dominance is consistent with the ecological conditions of the small intestine, including rapid digesta transit, exposure to bile acids, and relatively low bacterial density compared with the cecum (Oakley et al., 2014; Clavijo and Flórez, 2018).

The more pronounced response observed in the cecum likely reflects the distinct ecological role of this intestinal segment as the principal site of microbial fermentation in poultry. At the taxonomic level, GOS administration resulted in reduced relative dominance of *Lactobacillus* and increased abundance of fermentative taxa, including *Faecalibacterium, Blautia*, unclassified members of the families Lachnospiraceae and Ruminococcaceae, as well as *Barnesiella* and *Sporobacter*. These taxa predominantly belong to the class Clostridia and the phylum Bacteroidota, which include bacterial groups commonly associated with the degradation of complex polysaccharides and the production of SCFAs (Bortoluzzi et al., 2017; Wu et al., 2018). The increased abundance of *Faecalibacterium*, a major butyrate-producing genus, may suggest a greater representation of bacteria associated with butyrate production. Similarly, the expansion of members of the families Ruminococcaceae and Lachnospiraceae may indicate increased representation of bacterial groups involved in polysaccharide fermentation and SCFAs.

Although no significant differences in overall cecal community structure were detected by PERMANOVA, differential abundance analyses identified several taxa associated with GOS treatment. Notably, *Fusicatenibacter* was consistently detected by both LEfSe and ANCOM-BC2, while LEfSe additionally identified *Barnesiella, Sporobacter*, and the *Christensenellaceae R-7 group* as discriminative taxa. These findings suggest that GOS influenced selected bacterial populations without inducing broad-scale restructuring of the cecal microbial community.

In the study by Yang et al. (2022), dietary GOS supplementation increased the abundance of *Faecalibacterium, Alistipes*, and *Bacteroides*, accompanied by alterations in cecal metabolite profiles (including lysophosphatidylcholines and amino acids) and regulation of genes involved in lipid metabolism. The authors demonstrated associations between cecal microbiota reorganization and changes in the metabolome and transcriptome of breast muscle. These findings suggest that shifts in specific bacterial populations may have functional consequences along the gut–metabolism axis. Metabolomic and transcriptomic analyses were not performed in the present study. Nevertheless, the expansion of these taxa may reflect changes in microbial community composition; however, direct assessment of microbial metabolism was beyond the scope of the present study. In contrast, Richards et al. (2020) showed that dietary GOS supplementation in juvenile broilers shifted *Lactobacillus* populations (including increased abundance of *L. johnsonii*) and was associated with altered intestinal cytokine expression, characterized by upregulation of *IL-17A* and downregulation of *IL-10*. The authors proposed that GOS-driven microbiota modulation may program innate intestinal immunity and contribute to improved production performance.

Within the context of the present study, the biological significance of the observed taxonomic shifts and increased diversity in the cecum remains unclear. Further studies incorporating functional and immunological analyses are required to determine whether these microbial changes are associated with metabolic or immunological outcomes. The ileum responded primarily with increased richness while maintaining *Lactobacillus* dominance, whereas the cecum – being the principal fermentative chamber – exhibited changes in the relative abundance of several fermentative taxa.

Overall, the results indicate that *in ovo* administration of GOS induced segment-specific changes in gut microbial communities observed at day 42 of age. Whether these microbial changes are associated with alterations in host metabolism or immune function remains to be determined through dedicated functional studies.

### Effect of sodium butyrate on ileal and cecal microbiota

SB induced marked taxonomic changes in the ileal microbiota, indicating a stronger microbiota-modulating effect than that observed for GOS. Analysis of the relative abundance of dominant taxa demonstrated a reduced contribution of Lactobacillaceae (primarily *Lactobacillus*) in the SB group, accompanied by an increased proportion of taxa belonging to the class Clostridia, including members of the families Lachnospiraceae and Ruminococcaceae, as well as genera such as *Faecalibacterium, Butyricicoccus, Anaerobutyricum*, and *Gemmiger*. In addition, a higher relative abundance of Bacteroidota representatives, including *Bacteroides* and *Barnesiella*, was observed. LEfSe analysis confirmed a broad spectrum of discriminant taxa in the SB group, encompassing both Clostridia-affiliated taxa and Bacteroidota. Importantly, several of the taxa identified by LEfSe, including *Faecalibacterium, Bacteroides, Ligilactobacillus, Merdicola, Anaerobutyricum*, and unclassified Acutalibacteraceae, were also confirmed by ANCOM-BC2, supporting the robustness of the SB-associated microbial signature in the ileum.

In contrast, Yang et al. (2018), who investigated dietary supplementation with glycerol monobutyrate, did not observe significant changes in alpha or beta diversity in the ileum, and taxonomic shifts were limited. Several factors may account for the discrepancies between studies, including differences in the route of administration, the age of birds at sampling, and the timing of microbial modulation during early development. It is plausible that modulation of the intestinal environment at the embryonic stage may promote more persistent structural alterations; however, this hypothesis requires further verification. The increased abundance of SCFA-producing taxa in the ileum indicates a greater representation of bacterial groups commonly associated with fermentative metabolism. Nevertheless, functional interpretation based solely on taxonomic data remains indirect and would require confirmation through metabolomic measurements.

The changes observed in the cecum likely reflects the unique ecological characteristics of this intestinal segment, which serves as the principal site of microbial fermentation in poultry. Analysis of the relative abundance of the twenty most abundant taxa revealed a shift from Lactobacillaceae dominance toward increased representation of Clostridia and Bacteroidota. The rise in relative abundance included *Faecalibacterium, Butyricicoccus, Anaerobutyricum, Gemmiger*, as well as *Bacteroides, Barnesiella*, and *Alistipes*. LEfSe analysis further confirmed overrepresentation of Clostridia-affiliated taxa as discriminant markers of the SB group. Although no significant differences in overall cecal community structure were detected, both LEfSe and ANCOM-BC2 consistently identified several Clostridia-affiliated taxa, including *Butyricicoccus, Anaerobutyricum*, unclassified *Lachnospiraceae*, and unclassified *Acutalibacteraceae*, as characteristic of the SB group. This pattern suggests selective enrichment of fermentative bacterial populations rather than broad-scale restructuring of the cecal microbial community. *Faecalibacterium spp.* is recognized as a butyrate producer and an important component of fermentative microbial consortia, although its immunomodulatory properties have been predominantly described in mammalian models. Therefore, the increased abundance of SCFA-associated taxa may suggest altered fermentative potential of the cecal microbiota; however, in the absence of direct metabolomic data, such functional inferences remain indirect.

The direction of taxonomic changes observed in the present study is consistent with findings by Akram et al. (2024), who also reported increased representation of fermentative taxa from Clostridia and Bacteroidota following *in ovo* SB administration (0.3%), although they observed elevated Shannon and Simpson indices. Deng et al. (2023) and Melaku et al. (2024) described significant structural shifts in cecal microbiota without changes in alpha diversity. Wu et al. (2018) reported distinct clustering patterns accompanied by increased *Lachnospiraceae* and *Ruminococcaceae* and reduced *Enterobacteriaceae* and, under certain conditions, *Lactobacillaceae*. Similar trends were noted by Bortoluzzi et al. (2017) and Xiao et al. (2023), who documented enrichment of *Ruminococcaceae, Lachnospiraceae,* and SCFA-producing taxa following butyrate supplementation.

Collectively, available evidence suggests that SB consistently promotes the enrichment of cecal bacterial taxa commonly linked to fermentative processes. The direction of alpha diversity changes (increase, no change, or decrease) appears context-dependent, influenced by biological model, age of birds, and route or formulation of administration. The reduction in alpha diversity observed in the present study may reflect selective dominance of functionally specialized taxa rather than ecosystem destabilization; nevertheless, this interpretation requires confirmation through functional analyses.

Comparison of the effects of GOS and SB indicates that, although both interventions promoted bacterial taxa commonly associated with fermentative metabolism, the resulting patterns of microbiota modulation differed. GOS was associated with expansion and increased evenness of the bacterial community, particularly in the cecum, while maintaining relative structural stability in the ileum. In contrast, SB was associated with a broader range of differentially abundant taxa, particularly within Clostridia, together with a segment-specific impact on diversity parameters.

These findings suggest that modulation of the gut microbiota by a prebiotic (GOS) and a postbiotic (SB) results in distinct bacterial ecosystem configurations, and that the microbial response is determined both by the nature of the intervention and by the anatomical compartment of the gastrointestinal tract.

## Conclusions

*In ovo* administration of GOS and SB was associated with distinct microbiota profiles in the ileum and cecum of broiler chickens at day 42 of age. GOS promoted increased diversity and evenness of the bacterial community, particularly in the cecum, whereas SB was associated with stronger taxonomic shifts, including enrichment of Clostridia-affiliated taxa and reduced cecal diversity. Discriminant analysis further identified characteristic microbial signatures associated with both interventions. These findings indicate that prebiotic and postbiotic administration *in ovo* may differentially shape intestinal microbial communities and represent a promising strategy for microbiota-targeted interventions in poultry. Nevertheless, further studies are required to determine the functional consequences of these microbial changes and to evaluate their relevance under commercial production conditions.

## Author contribution

*S. Knaga:* Writing – original draft, Investigation, Methodology, Data curation.*M.Z. Akram:* Formal analysis, Software, Data curation, Visualization, Validation, Writing – review & editing.*A. Bełdowska-Krężel:* Investigation, Methodology, Resources.*E. Pietrzak:* Investigation, Resources.*A. Dunisławska:* Conceptualization, Methodology, Supervision, Project administration, Resources, Writing – review & editing.

## Disclosures

The authors declare that they have no known competing financial interests or personal relationships that could have appeared to influence the work reported in this paper.
